# Color polarization vision mediates the strength of an evolutionary trap

**DOI:** 10.1111/eva.12690

**Published:** 2018-09-24

**Authors:** Bruce A. Robertson, Gábor Horváth

**Affiliations:** ^1^ Division of Science, Mathematics and Computing Bard College Annandale‐on‐Hudson New York; ^2^ Environmental Optics Laboratory Department of Biological Physics, Physical Institute ELTE Eötvös Loránd University Budapest Hungary

**Keywords:** aquatic insect, behavior, color preference, color sensitivity, evolutionary trap, light pollution, maladaptation, polarized light pollution

## Abstract

Evolutionary traps are scenarios in which animals are fooled by rapidly changing conditions into preferring poor‐quality resources over those that better improve survival and reproductive success. The maladaptive attraction of aquatic insects to artificial sources of horizontally polarized light (e.g., glass buildings, asphalt roads) has become a first model system by which scientists can investigate the behavioral mechanisms that cause traps to occur. We employ this field‐based system to experimentally investigate (a) in which portion(s) of the spectrum are polarizationally water‐imitating reflectors attractive to nocturnal terrestrial and aquatics insects, and (b) which modern lamp types result in greater attraction in this typical kind of nocturnal polarized light pollution. We found that most aquatic taxa exhibited preferences for lamps based upon their color spectra, most having lowest preference for lamps emitting blue and red light. Yet, despite previously established preference for higher degrees of polarization of reflected light, most aquatic insect families were attracted to traps based upon their unpolarized spectrum. Chironomid midges, alone, showed a preference for the color of lamplight in both the horizontally polarized and unpolarized spectra indicating only this family has evolved to use light in this color range as a source of information to guide its nocturnal habitat selection. These results demonstrate that the color of artificial lighting can exacerbate or reduce its attractiveness to aquatic insects, but that the strength of attractiveness of nocturnal evolutionary traps, and so their demographic consequences, is primarily driven by unpolarized light pollution. This focuses management attention on limiting broad‐spectrum light pollution, as well as its intentional deployment to attract insects back to natural habitats.

## 1 INTRODUCTION

Animals use light as a source of information to guide behaviors such as orientation (Lorne & Salmon, 2007), reproduction (Rand, Bridarolli, Dries, & Ryan, 1997), and foraging (Dwyer, Bearhop, Campbell, & Bryant, 2013). Some of this light is polarized, meaning that the direction of the electric field vector of light is oriented nonrandomly, such that it vibrates predominantly in some direction (e.g., horizontally). Sky polarization patterns may be used by animals as a cue for orientation (Dacke, Nilsson, Scholtz, Byrne, & Warrant, 2003; Muheim, Sjöberg, & Pinzon‐Rodriguez, 2016), but the predominant source of polarized light on the earth's surface is water bodies (Horváth, Kriska, Malik, & Robertson, 2009). When sun‐ or moonlight reflects from water surfaces, it typically becomes horizontally polarized with degrees of polarization (*d*) depending on the darkness/brightness of the water body and on the angle of reflection (Horváth et al., 2009). Historically, water‐seeking aquatic insects have reliably used the horizontal polarization of light reflected with a high enough degree of polarization *d* to locate water sources they require to feed, mate and lay eggs (reviewed in Horváth & Csabai, 2014). Today, it is well‐known that the abundance of smooth, dark‐colored, man‐made objects (e.g., asphalt, glass buildings, black cars, solar panels: *d* ≈ 80%–100% at and near the Brewster's angle θ_Brewster_ = arctan *n* measured from the normal vector of the reflecting surface with refractive index *n*; Kriska, Horváth, & Andrikovics, 1998; Kriska, Csabai, Boda, Malik, & Horváth, 2006; Kriska, Bernáth, Farkas, & Horváth, 2009; Horváth et al., 2009, 2010) can polarize light more strongly (i.e., with higher *d*) than any natural water body (*d* ≈ 30%–80%, Flamarique & Browman, 2001; Horváth, 2014) and water‐seeking aquatic insects are preferentially attracted to oviposit upon these supernormally intense sources of horizontal polarization where their eggs fail to hatch (Horváth et al., 2010; Kriska et al., 1998, 2006, 2009). Adults then die, typically with no second opportunity to reproduce. Such cases of anthropogenic change where evolved cue‐resource correlations are uncoupled and animals come to prefer inferior habitats are known as ecological traps (Dwernychuk & Boag, 1972), which are one type of the broader category of evolutionary traps in which any type of behavior is similarly affected (Schlaepfer, Runge, & Sherman, 2002). These severe forms of behavioral maladaptation are being detected at an increasing frequency (Robertson & Hutto, 2006; Robertson, Rehage, & Sih, 2013), are known to lead to severe and rapid population declines (Delibes, Ferreras, & Gaona, 2001; Fletcher, Orrock, & Robertson, 2012; Kokko & Sutherland, 2001), and affect a broad range of insect taxa (Robertson et al., 2013; Robertson, Campbell et al., 2017; Robertson et al., 2018).

When placed above water bodies or artificial polarizers, lamps create both polarized (due to reflected light) and unpolarized (by direct light) photopollution that can, individually or in combination, trigger maladaptive behavior in a diverse range of insect taxa (Boda, Horváth, Kriska, Blahó, & Csabai, 2014; Robertson, Campbell et al., 2017; Száz et al., 2015). Yet, species differ widely in their susceptibility to these different forms of light pollution (Robertson, Campbell et al., 2017), and the specific mechanisms by which these man‐made light sources interface with evolved behavioral rules to trigger maladaptation remain poorly understood (Hölker et al., 2010; Horváth et al., 2009; Longcore & Rich, 2004; Perkin, Hölker, & Tockner, 2014; Robertson et al., 2013). Artificial light sources (e.g., light‐emitting diodes—LEDs, high‐pressure sodium [HPS] lamps) differ from sunlight and moonlight in that different lamp types emphasize, or entirely omit, particular wavelengths of light (Gaston, Visser, & Hölker, 2015). When it reflects from a man‐made object, lamp light will not be polarized evenly across the visible spectrum. Wavelengths reflected with highest intensity are polarized the least, that is, with lowest degree of polarization (Hegedüs & Horváth, 2004). In contrast, apart from the case when the sun and moon are close to the horizon, sun‐ and moonlight are relatively uniform in intensity across visible wavelengths (Gaston et al., 2015), and so polarization of natural light via reflection from colorless (i.e., black, grey or white) water bodies will result in a similarly almost uniform degree of polarization across the visible spectrum. Collectively, this means that by emphasizing particular wavelengths of light, artificial light pollution has the evolutionarily novel effect of decoupling the historically correlated relationship between the intensity of light and the degree of polarization of water‐reflected light. It also means that if night‐active water‐seeking flying insects disproportionately rely upon different horizontally polarized portions of the visible spectrum to locate water bodies, artificial lighting located near artificial polarizers (e.g., asphalt, glass buildings) could make those objects more attractive to nocturnal ovipositing insects than actual water bodies.

In this way, lamps that emit different spectra of unpolarized light have the capability of exacerbating the severity of ecological traps caused by nocturnal polarized light pollution or to reduce their demographic impacts, because stronger habitat preferences trigger ecological traps with more severe demographic consequences (Fletcher et al., 2012; Hale & Swearer, 2016; Robertson et al., 2013). Global‐scale shifts toward broader spectrum street lighting (e.g., LEDs) for their energy efficiency, and safety benefits will result in shifts in the available wavelengths of nocturnal polarized light across large landscapes with potential to exacerbate or mitigate maladaptive behavioral responses in water‐seeking insects.

We designed a field experiment to ask (a) whether nocturnal water‐seeking aquatic insects rely upon different spectra of polarized light to locate water bodies and (b) which lamp types (and portions of their visible spectra) insects are preferentially attracted to and so which sensory cues mitigate or exacerbate the severity of evolutionary traps triggered by nocturnal polarized light pollution. Our approach examines variation in riverside captures of seven aquatic and six terrestrial night‐active insect families in simulated water bodies (white and black oil‐filled trays) illuminated by LED, metal‐halide (MH), or HPS lamps. We interpret differentially higher captures among lamp types illuminating oil‐filled horizontal white trays (full‐spectrum reflectors of unpolarized light) as an attraction to a particular spectrum of unpolarized light produced by that lamp and reflected by the tray. Differential attraction to a particular lamp treatment illuminating black trays (full‐spectrum reflectors of horizontally polarized light) is interpreted as evidence of preferential attraction to particular spectra of horizontal polarization. Interactions between tray and lamp types suggest preferential attraction to horizontally polarized light of a spectrum that differs from the preferred spectrum of unpolarized light and *vice versa*, indicating independence of detection of the wavelength and polarization of reflected light. Terrestrial taxa are commonly attracted to bright, unpolarized lights (Nowinszky, 2003; Nowinszky, Kiss, Szentkirályi, Puskás, & Ladányi, 2012), and empirical studies suggest their ventral eye region does not perceive the polarization of light coming from below (reviewed in Horváth, 2014; Robertson, Campbell et al., 2017), but evidence is limited. We examine the behavioral responses of terrestrial taxa to polarized light treatments to confirm these negative responses at our study sites and so contextualize the impacts of polarized and unpolarized light treatments on aquatic insects.

## 2 MATERIALS AND METHODS

### 2.1 Study sites and experimental design

**Figure 1:**
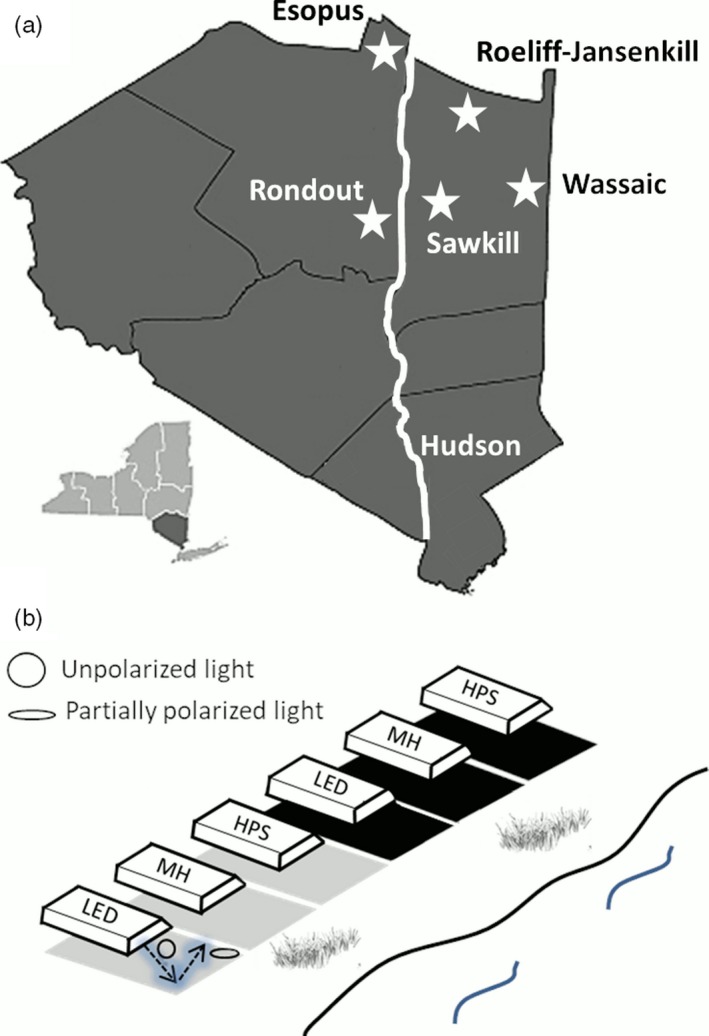
a) Locations of field experiments in the Hudson Valley region of southern New York State. Stars indicate locations of experimental sites on five tributaries of the Hudson River (in white) located in two of the six counties outlined in black. b) The spatial arrangement of oil‐filled black and white trays illuminated by 1) high‐pressure sodium (HPS) lamp, 2) light‐emitting diodes (LED), and 3) metal‐halide (MH) lamp at each study site. Oil‐filled trays of two colors reflect lamplight and polarize it to a greater (black) or lesser (white) degree and are placed 5 meters from the edge of study streams

We selected study sites on five different tributaries of the Hudson River in southern New York State, USA. (Figure 1a). Study sites were identified in sparsely populated areas along heavily forested river corridors. We chose residential properties that maintained mown lawns extending from the high water line inland at least 60 m to ensure sufficient area for our experiment and so that vegetation would not impede insects’ lines of sight or movement toward experimental test surfaces. In 2013, we trapped flying emergent aquatic insects two times at each site: Visit 1) June 17–July 6; and Visit 2) July 10–24. Each trapping session lasted for 120 min, beginning exactly 30 min after sunset.

**Figure 2:**
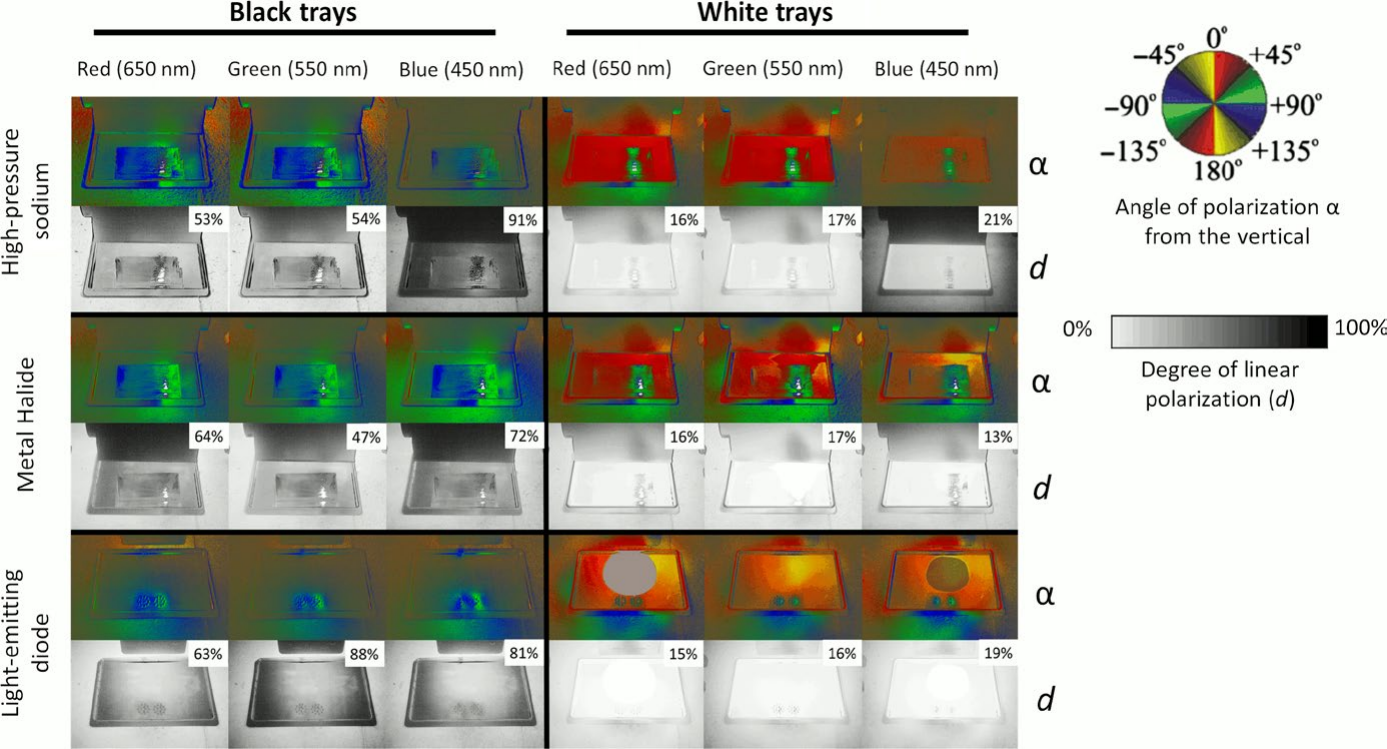
Reflection–polarization characteristics (angle α measured clockwise from the vertical and degree *d* of linear polarization of reflected light) of salad‐oil‐filled black (left) and white (right) trays illuminated by high‐pressure sodium (HPS) (top), metal‐halide (center), and light‐emitting diode (bottom) lamp used in the choice experiments, measured with imaging polarimetry in the red (650 nm), green (550 nm), and blue (450 nm) portions of the spectrum. White trays reflected lamplight with much lower degrees of polarization than black trays. The maximum *d* value is given for each tray. HPS light was reflected with the highest *d* in the blue, reflected LED light was most strongly polarized in the green, and MH lamplight was polarized to a high and more similar degree across the red, green, and blue spectra. Black trays consistently reflected horizontally polarized light. White trays reflected primarily vertically polarized light with the exception of horizontal polarization of the mirror image of the light source

**Figure 3:**
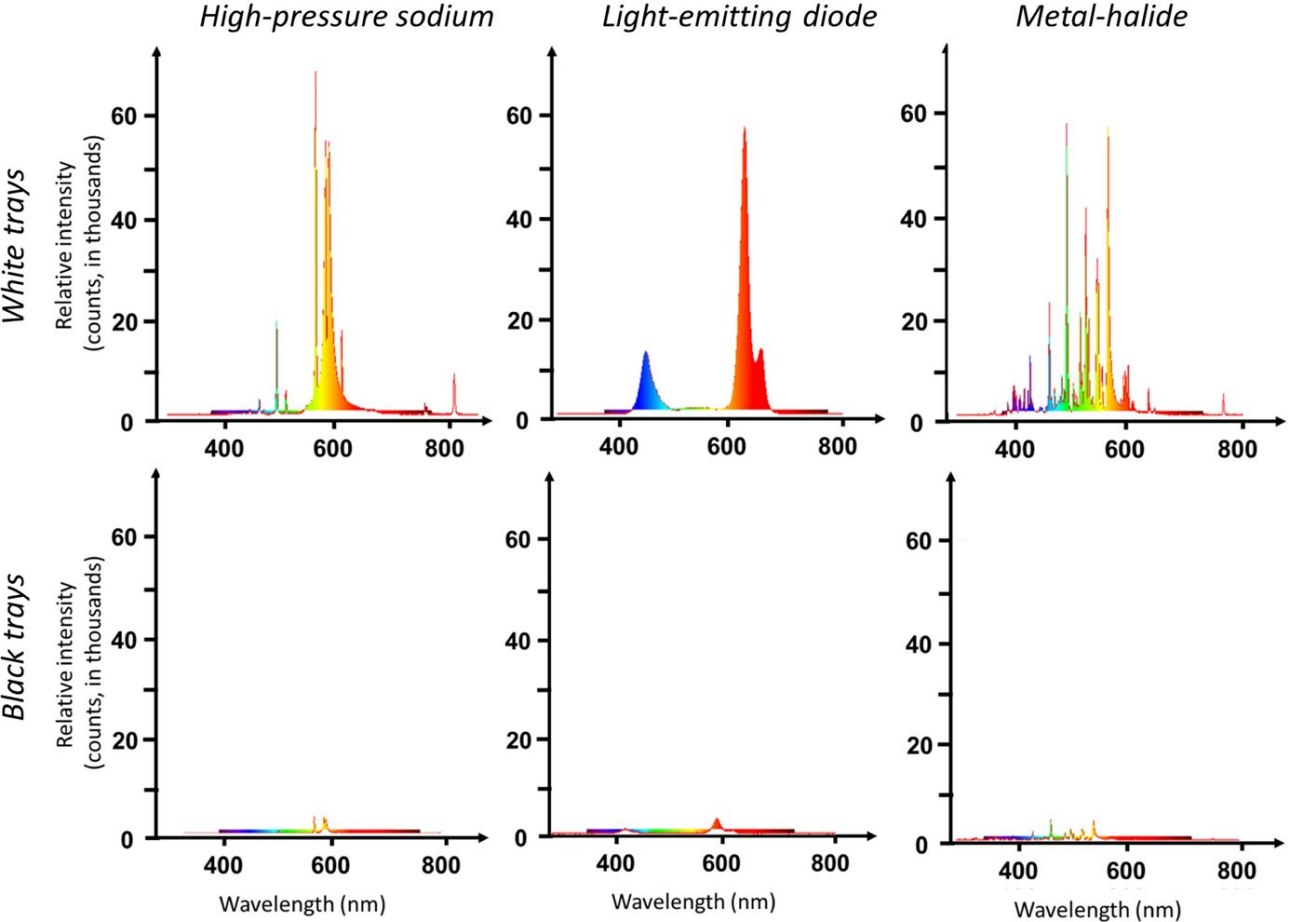
Spectra of light reflected from the white (top row) and black (bottom row) trays illuminated by three different types of nocturnal illumination: high‐pressure sodium lamp, light‐emitting diodes, and metal‐halide lamp. We measured the relative irradiance of light entering the cosine‐correcting sensor of a spectrometer 4 cm above the geometric center of the tray and aiming downward at 45^o^ in the direction of the mirror image of the lamp above

We used black and white trays (60 × 40 cm) filled with transparent common salad oil to capture insects and assess their relative preference for test surfaces varying in their a) illumination type and b) degree of polarization of reflected light (*d*, Figures 1b and 2). We placed three of each of the two tray colors (black, white) in a row parallel to the riverbank at a distance of 5 meters, spaced 0.5 m apart. Above each of the trays, we suspended a 180‐watt array of LED, a 150‐watt HPS bulb, or a 150‐watt MH bulb (Figure 1b). We used an Ocean Optics USB2000+ spectrometer to measure the absolute irradiance (μW/cm^2^/nm) of each light source as a function of wavelength. We selected lamps of wattages that produced similar overall luminosity (11,000–12,000 lumens) among lamp types, though, at different wavelengths (Figure 3). We chose a relatively narrowband (yellow/green: 550–630 nm) HPS source, a LED source that peaks at two complementary ranges of wavelengths (blue: 420–480 nm; red: 620–680 nm) with red wavelengths being more common at dawn and dusk, and a MH lamp that produces light at nearly all visible wavelengths (Figure 3). Each light source was placed inside a downward facing 35 × 54 cm aluminum reflector painted black on top to minimize its visual signature and elevated 30 cm above the test surface. In this way, there were two treatments of each lamp type illuminating both white and black trays. Lamps were hung low above test surfaces (25 cm) and shaded to illuminate the test surfaces such that insect responses would be based upon their perception of only tray‐reflected (not direct) light, except at very short range (after Robertson, Campbell et al., 2017).

We exposed these six combinations of lamp spectra and tray color treatments to aquatic insects during each of the two visits to each of the five study sites. Adjusted (see Statistical Analysis, below) captures from each of the two sampling sessions at a study site were combined prior to analysis, leading to a total sample size of *n* = 30 for the study. We randomized the location of trays and lamp types in our study to avoid bias in captures associated with the physical position of treatments in the experiment relative to the surrounding environment and the other treatments. The spatial arrangement of treatment combinations was randomized at each site visit, with the exception that light sources of the same type were never placed immediately next to each other. The positions of the white and black trays within a lighting treatment were exchanged every 20 min. After each sampling session, we poured tray contents through fine cheesecloth to separate insects, which were stored in 80% ethanol for later identification to the family level.

### 2.2 Focal taxa and experimental predictions

We define aquatic insect taxa as those with a larval or adult life‐history phase dependent on the availability of a freshwater source (Johnson & Triplehorn, 2004). We focused our analysis on aquatic insect taxa known to exhibit stronger attraction to sources of horizontally polarized light with greater *d*‐values: Ephemeroptera (Horváth et al., 2010; Kriska et al., 1998, 2006; Száz et al., 2015), Trichoptera (Horváth et al., 2010; Kriska, Malik, Szivák, & Horváth, 2008), Empididae (Robertson et al., 2018), Simuliidae (Robertson et al., 2018), and Chironomidae (Horváth, Móra, Bernáth, & Kriska, 2011; Lerner et al., 2008, 2011; Robertson et al., 2018). When seeking a suitable oviposition site, these taxa touch down on the surface of water bodies (Horváth, 2014; Kriska et al., 2006), but will be captured in the oil traps, because oil wets chitin, preventing take off.

### 2.3 Polarizing properties of traps

We measured the reflection–polarization characteristics of oil‐filled trays using imaging polarimetry (Horváth & Varjú, 2004) in the red (650 ± 40 nm = wavelength of maximal sensitivity ± half bandwidth of the polarimeter detector), green (550 ± 40 nm), and blue (450 ± 40 nm) portions of the spectrum. We performed polarimetric measurements in a dark room with the optical axis aimed downward with the reflector (oil‐filled trays) aimed at the Brewster's angle θ_Brewster_ = arctan (*n *= 1.5) = 56.3° from the vertical calculated for the refractive index *n* = 1.5 of vegetable oil. Exposures were standardized for 1/30th of a second at 400 ISO. At the Brewster's angle, surface‐reflected light is perpendicular to the refracted ray penetrating the oil, resulting in the highest possible degree of polarization. Three images were taken of each object with the polarization filter at different angles. Those images were processed via Polarworks© software into images illustrating the degree (*d*) and angle (α) of polarization at each pixel. We estimated the maximum degree of polarization associated with trays by quantifying the fraction of the *d*‐patterns composed of black pixels using ImageJ© software. We calculated a window size representing 1/10 of the tray length and height and estimated maximum *d* as the window giving the maximum value out of 20 nonoverlapping sample locations within the image of each tray nonrandomly focused on portions of the tray with the highest visual estimates of *d* on the black‐to‐white scale.

### 2.4 Statistical analyses

We examined the effect of 1) lamp type and 2) the *d* (degree of polarization of reflected lamplight) on the number of captured individuals within each insect family. Family‐specific analyses serve to illustrate the behavioral preferences of each taxon. For these analyses, we created a standardized abundance metric that would be reflective of the relative fraction of individuals attracted to each treatment and that was insensitive to bias associated with disproportionately high or low captures at one study site relative to others. We adjusted captures of each family associated with each test surface to be a fraction of the total number of captures for that visit at that site, then multiplied each fraction by 100, rounding to the nearest whole individual. We modeled standardized captures using generalized linear models and fit models to negative binomial or Tweedie distributions with a log‐link function. Tweedie is a family of probability distributions, which have positive mass at zero, but are otherwise continuous and include the Poisson distribution which is typical of count data. Negative binomial distributions are also commonly used to model count data. We modeled lamp and tray type as categorical variables, in predicting captures for each family and selected the error distribution model that minimized overdispersion (ĉ).

## 3 RESULTS

### 3.1 Polarization imagery

Black trays were more efficient polarizers of reflected light than white ones (Figure 2). The black test surfaces reflected light with very high degrees of polarization (*d*_black_ = 53%–88%), including values higher than water is generally capable of (*d*_water_ = 30%–80%, Horváth & Varjú, 2004; Horváth, 2014). White trays reflected very low degrees of polarization (*d*_white_ = 15%–21%). Black test surfaces reflected always horizontally polarized light (because the horizontally polarized light reflected from the air–water interface was dominating), while white test surfaces reflected mainly vertically polarized light (because the vertically polarized light reflected from the tray's bottom and refracted at the water–air interface was dominating). The small mirror image of light sources reflected by the white trays was horizontally polarized (because there again the horizontally polarized light reflected from the water surface dominated) with maximum *d*‐values (Figure 2).

Black trays reflected light with lower and higher *d* in those spectral ranges in which the intensity of illuminating lamplight was higher and lower, respectively (Figures 2 and 3): HPS light reflected by the black test surfaces was most polarized in the blue range (Figure 2) where its unpolarized intensity is minimal (Figure 3). MH lamps emitted maximal intensity in the green spectral range (Figure 3), in which therefore the *d* of light reflected from the black trays was minimal (Figure 2). Contrary to the latter lamp type, the LEDs used were less intense in the green (Figure 3); thus, *d* of LED light reflected by the black test surfaces was maximal in the green (Figure 2).

### 3.2 Behavioral responses of arthropods

We captured a total of 75,992 adult aquatic insects in 27 families. We focused our analysis on 13 families for which we were able to fit models without overdispersion (ĉ < 4.0, all *df* = 30, Table 1). These included seven families of aquatic insects (Caenidae, Chironomidae, Empididae, Glossosomatidae, Heptageniidae, Hydropsychidae, and Isonychiidae) and six families of terrestrial insects (Cecidomyiidae, Cercopidae, Cicadellidae, Scarabaeidae, Tipulidae, and Geometridae). Hydropsychid and isonychid data were fit using models with a Tweedie distribution, while the remaining families were fit with negative binomial distributions. Fit of data to models was good (ĉ = 0.95–3.91).

**Table 1:** Abundance model output information relative to the seven most abundant families of emergent aquatic insects and six families of terrestrial insects with no aquatic life‐history stage captured in the field experiments

Family	Common name	No. of individuals	% of captures	Model	*χ* ^2^ values		
					Lamp	Tray	Interaction
Aquatic							
Caenidae	Squaregill mayflies	889	1.1%	NB	6.95*	4.07*	
Chironomidae	Nonbiting midges	14,465	18.4%	NB	10.78**	58.64***	54.69***
Empididae	Dance flies	3,622	4.6%	NB	2.65	9.35**	
Glossosomatidae	Saddle‐case mayflies	4,955	6.3%	NB	9.13*	4.80*	
Heptageniidae	Flat‐headed mayflies	28,375	36.2%	T	7.22*	0.08	
Hydropsychidae	Net‐spinning caddisflies	16,010	20.4%	T	23.10***	10.33**	
Isonychiidae	Brush‐legged mayflies	1,187	1.5%	T	8.54*	0.91	
Terrestrial							
Cecidomyiidae	Gall midges	5,279	6.7%	NB	0.58	2.80	
Cercopidae	Froghoppers	652	0.8%	NB	4.95	1.26	
Cicadellidae	Leafhoppers	2,624	3.3%	NB	0.52	2.81	
Scarabaeidae	Scarab beetles	222	0.3%	NB	0.90	2.61	
Tipulidae	Crane flies	84	0.1%	NB	3.99	3.60	
Geometridae	Inchworm moths	85	0.1%	NB	28.70***	0.78	

*Notes*

Captures of individuals in these groups were modeled as functions of lamp and tray type. Data were fit to either negative binomial (NB) or Tweedie (T) distributions.

Wald chi‐squared values associated with each independent variable are given along with their statistical significance (*p < 0.05; **p < 0.01; ***p < 0.001).

**Figure 4:**
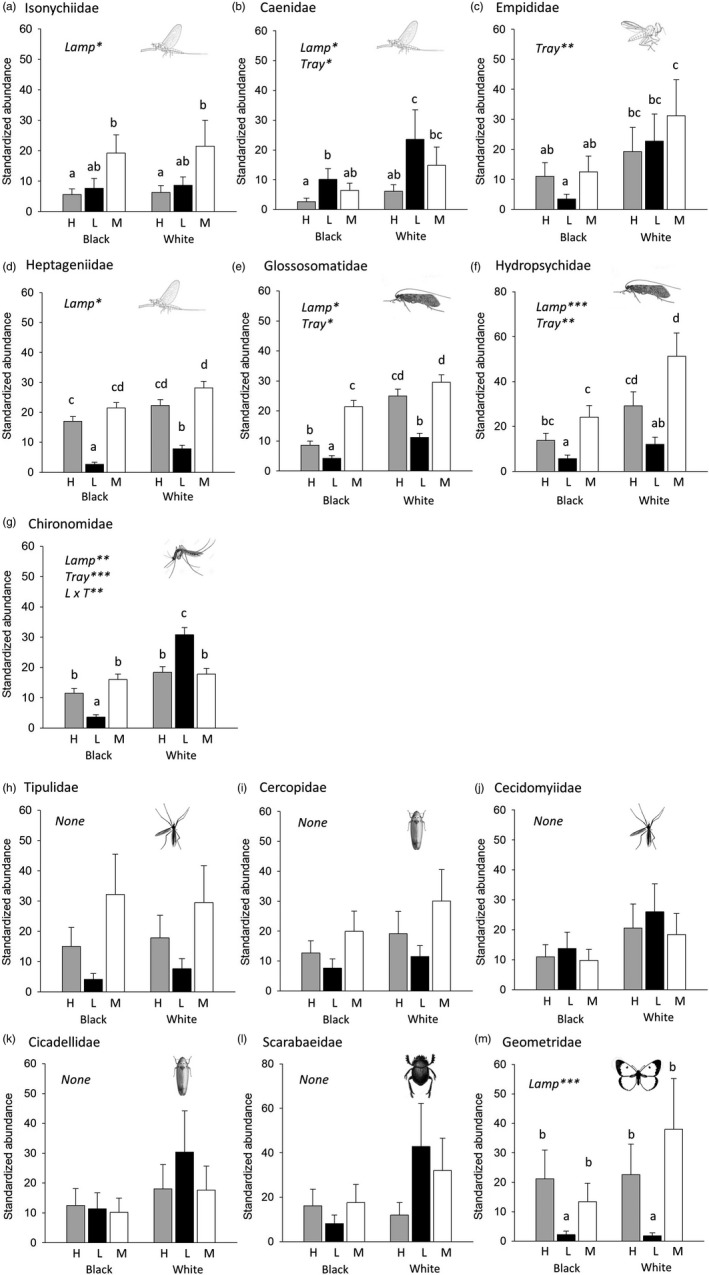
Standardized abundance of insect families captured by the differently illuminated oil traps as a function of the type of illumination (Lamp) and the color (black or white) of the insect trap (tray). Black and white oil‐filled trays were placed below downward‐facing lamps of three types: high‐pressure sodium, light‐emitting diode, and metal‐halide (M, see Figure 1). Black trays strongly polarize the visible wavelengths as would dark lakes and other dark water bodies. Standardized abundance is the relative number of captures per treatment (± standard error) for each family of aquatic (a‐g) and entirely terrestrial (h‐m) insects. Statistical significance of independent variables is shown in each figure: *p < 0.05; **p < 0.01; ***p < 0.001 (see Table 1). Letters a, b, and c above each treatment represent results of pairwise contrast post hoc tests such that columns that do not share the same letter represent means that are different at the *p* = 0.05 level. Images are typical morphological representations of each taxonomic group

All seven aquatic families exhibited a preference for lamp and/or tray type (Figure 4, Table 1). Isonychid mayflies were captured in progressively greater numbers in LED and MH lamps than sodium lamps (Figure 4a), while caenid mayflies exhibited a preferential attraction to LEDs and to white test surfaces in general (Figure 4b). Dance flies (Empididae) exhibited preference only for white test surfaces, regardless of lamp type (Figure 4c). Flat‐headed mayflies (Heptageniidae) were captured in lowest abundance in LED treatments (Figure 4d). Glossomatid and hydropsychid mayflies were also captured in lowest numbers in LED‐illuminated traps, but were also more attracted to white test surfaces (Figures 4e,f). Nonbiting midges (Chironomidae) were differentially attracted to LEDs associated with white test surfaces while exhibiting lowest preference for them when they were associated with black ones (Figure 4g). Among terrestrial insects, only one family exhibited differential attraction to an experimental treatment: Geometrid moths were attracted to sodium and MH lamps relative to LEDs (Figure 4m).

## 4 DISCUSSION

The reduced intensity of photons available at night has been thought to make the process of color vision difficult and so select against its use (Warrant & Dacke, 2011). Yet, the spectral composition of artificial light is known to affect degree of attraction by both terrestrial and aquatic insects (van Grunsven et al., 2014; Longcore et al., 2015; Pawson & Bader, 2014), and we found color preference was ubiquitous among the night‐active aquatic insects we studied. This study represents the first experimental investigation of the role of spectral preference in triggering nocturnal evolutionary traps induced by polarized‐light‐polluting artificial reflectors. We found that aquatic insect families exhibited distinct and maladaptive preferences for specific wavelengths of both unpolarized light and polarized light reflected from black or white test surfaces that have implications for their conservation and the management of light pollution.

Color vision and preference are known among diurnal (Hoback, Svatos, Spomer, & Higley, 1999; Radwell & Camp, 2009) and crepuscular (Schwind, 1991, 1995) aquatic insects. We found spectrum‐dependent phototaxis in nocturnal aquatic insects to fall into four categories: (a) Attraction to MH lamps (Isonychiidae), (b) attraction to LED lamps (Caenidae), (c) preference for MH and HPS lamps relative to LEDs (Heptageniidae, Glossosomatidae, Hydropsychidae), and (d) polarization‐dependent preference relative to blue‐red LEDs (Chironomidae). Broad‐spectrum MH lamps were the most attractive lamp type for five of the seven families. The spectra of the specialized blue‐red LEDs we used in this study were found consistently most attractive only by a family of tiny mayflies (Caenidae) known from previous studies to find these two colors relatively unattractive (Radwell & Camp, 2009). Other mayflies in this study preferred MH and HPS lamplight over blue‐red LEDs, which is the more typical behavior of diurnal Ephemeroptera (Hoback et al., 1999; Radwell & Camp, 2009). Five of the six terrestrial insect families we examined exhibited no clear preference with respect to spectra. Exceptionally, geometrid moths were equally and more attracted to the HPS and MH lamps relative to LEDs. This group is known for its affinity for broad‐spectrum lighting and lamps emphasizing blue light (Barghini & de Medeiros, 2012; Bates et al., 2013; Lamphar & Kocifaj, 2013; van Langevelde, Ettema, Donners, Wallis‐DeVries, & Groenendijk, 2011; Somers‐Yeates, Hodgson, McGregor, Spalding, & ffrench‐Constant, 2013), but in this study were least attracted to LEDs, which emitted the widest diversity of blue wavelengths and at highest intensity. Whether the spectral preferences we identified originally evolved to guide animals during any diurnal or crepuscular activity periods is unclear. It is clear that these color preferences are currently guiding nocturnal behavior and could represent adaptations designed to guide nocturnal habitat selection, navigation or mate selection and so may be adaptive, non‐adaptive, or maladaptive in different behavioral context.

Only one of the seven aquatic, and none of the terrestrial insect families, in this study exhibited patterns of capture indicating differential attraction to reflected polarized light spectra. If insects in this study were exhibiting attraction for high degrees of polarization of reflected light independent of its color, they should have been captured more frequently in our water‐imitating traps associated with HPS and LED lamps, which produced the highest degrees of reflected polarization, but patterns of capture suggested that insects were not using polarized light to guide their habitat selection decisions. Instead, captures were commonly (five of the seven aquatic families) higher in treatments with nonpolarizing white test surfaces indicating that phototaxis was dominant over polarotaxis. Notably, these aquatic insect groups have been experimentally demonstrated to exhibit preference for water‐reflected light with higher degrees of polarization (Ephemeroptera: Kriska et al., 2009; trichoptera: Kriska et al., 2008; Lerner et al., 2008; Chironomidae: Robertson et al., 2018), yet ignore these habitat selection preferences when in the presence of unpolarized lamp light which simulates their primary navigational cue, the moon (Boda et al., 2014; Robertson, Campbell et al., 2017; Száz et al., 2015). Robertson, Campbell et al. (2017) concluded that this result indicates that a behavioral cue evolved to guide behavior in one context can be exploited to trigger an evolutionary trap in another. A more proximate explanation may be that polarization sensitivity is housed in the ommatidial cells that detect brightness in insect eyes (e.g., the brightness–polarization hypothesis; Labhart, 2016), much the same way that *Papilio* butterfly ommatidia contain both polarization and color sensitivity, leading to an inability to distinguish between these two modalities of light under artificial laboratory conditions (Hegedüs & Horváth, 2004; Kelber, 1999; Kelber, Thunell, & Arikawa, 2001).

Only chironomid midges exhibited patterns of captures consistent with wavelength‐specific preference for horizontally polarized light at night. Because unpolarized wavelengths produced at highest intensity by lamps are polarized the least by reflection (Hegedüs & Horváth, 2004; Figures 2 and 3), inference about evidence for color‐dependent polarization preference must also follow this rule. Previous studies indicated that day‐active chironomids are differentially attracted to unpolarized green/blue light (Burkett, 1998), but experimental studies of color preference in this group are rare. In our study, midges homed to the blue and/or red unpolarized LED light, but their reduced attraction to the horizontally polarized light treatment of this lamp type is actually consistent with preference for polarized light in this same spectrum. In accordance with the inverse relationship between intensity and degree of reflection–polarization, red/blue light was polarized the least, and green light the most by our LED lamp paired with the black polarizing reflector (Figure 2). And because patterns of chironomid capture are inconsistent with preference for treatment for the highest degree of polarization independent of color, we can infer that chironomids exhibit color‐dependent preference for red or blue horizontally polarized light. This result is surprising, because Schwind's (1991, 1995) studies show that all 18 species of day‐active aquatic insects he studied used specific spectra of polarized light to guide their habitat selection behavior.

Why might such wavelength‐specific polarization sensitivity or preference evolve? The first possibility is that lakes and rivers, or their bottoms, differ in their absorbance spectrum in ways that are tied to the survival of eggs and young. Shallow water bodies with abundant green plant or algal substrates, for example, reflect light with the highest degrees of horizontal polarization in the ultraviolet and blue ranges of the spectrum (Horváth & Varjú, 2004; Schwind, 1995). Because blue light can penetrate more deeply into natural clear water than UV and red light, preferences could reflect adaptation to the depth at which aquatic life‐stages live (Schwind, 1995). Alternatively, preferences for unpolarized light of different color in nocturnal insects may be a by‐product of the evolutionary tuning of light sensitivity to spectra associated with food (Hill, Wells, & Wells, 1997), refugia from predation, or conspecifics (Ellers, Boggs, & Mallet, 2003).

Nocturnal polarized light pollution (Horváth et al., 2009) differs fundamentally from its diurnal counterpart in that, in contrast to the full spectrum of sunlight, lamp spectra can be a constrained subset of wavelengths, but those that are produced differ dramatically in the intensity at which they are produced (Gaston et al., 2015). Thus, the degree of polarization of a reflector (natural or artificial) can be decoupled from the wavelength it has been associated with over evolutionary history, and the availability of wavelengths of light to be polarized can vary dramatically depending on the lamp itself. This represents a novel mechanism for the formation of an evolutionary trap. Finally, by capturing insects immediately upon touching the oil surface, our trapping method prevented insects from using olfactory cues to avoid unsuitable habitats, which may be more common in longer lived insects (Lerner et al., 2011).

Collectively, our results indicate that nocturnal aquatic insects are capable of color‐dependent polarization sensitivity that can exacerbate or mitigate their responses to light pollution depending on the spectrum of light pollution they are exposed to, and the qualities of the reflecting surfaces (e.g., buildings, asphalt) in the vicinity. Our results confirm that of previous studies (Boda et al., 2014; Robertson, Campbell et al., 2017; Száz et al., 2015) that nocturnal polarized light pollution is less important in triggering evolutionary traps than unpolarized lamplight, and indicate a partial trade‐off between spectrum intensity in inducing evolutionary traps via unpolarized vs. polarized light. Broad‐spectrum lamps that emphasize all wavelengths at relatively high intensity (e.g., some commercial LEDs) may have very weak polarized light signatures, but attract aquatic insects to their unpolarized spectra. Because reducing the attractiveness of evolutionary traps is the most important method to reduce their demographic impacts (Fletcher et al., 2012; Hale & Swearer, 2016; Robertson, Ostfeld, & Keesing, 2017; Robertson, Campbell et al., 2017), our results suggest that selection of more narrowband (especially blue‐red emphasizing) lamps by individuals and governments will have greater conservation benefits for more taxa of nocturnal aquatic insects than efforts to reduce the use of polarizing building and roadway materials near lakes and rivers.

Given the strong, negative fitness consequences that evolutionary traps have for individuals, natural selection against maladaptive behaviors should be strong, and so traps should be ephemeral in time (Schlaepfer, Sherman, Blossey & Runge 2005; Delibes et al. 2001). Indeed, a recent study illustrates the evolution of reduced sensitivity to unpolarized light pollution by urban moths (Altermatt & Ebert, 2016). Yet, they may persist even in the face of population collapse if use of polarized light to locate water or unpolarized light to navigate is canalized (Fletcher et al., 2012). The intentional construction of evolutionary traps to control pest species has recently been highlighted as a powerful new wildlife management tool (Horváth, Blahó, Egri, & Lerner, 2014; Robertson, Ostfeld et al., 2017), the principles of which can be adapted to manipulate native species exposed to evolutionary traps into making adaptive decisions (e.g., Horváth et al., 2010). For example, aquatic insects are attracted upward to the surfaces of bridges by unpolarized bridge‐lighting where they then oviposit on horizontally polarizing asphalt surfaces (Málnás et al., 2011), but downstream‐facing LEDs placed at the water surface can preferentially attract insects to oviposit on the water, instead (Egri et al., 2017). Because unpolarized light is primarily responsible for drawing aquatic insects away from suitable habitats, a similar strategy of placing a brighter or more broad‐spectrum lamp above water can be employed where residences or businesses expose lakes and streams to artificial light. Global trends toward the wholesale adoption of broad‐spectrum lamps for nocturnal illumination portend the expansion of evolutionary traps for both terrestrial and aquatic insects across the globe at a time when insect populations are already under collapse (Hallmann et al., 2017) and when continent‐scale evolutionary traps have been implicated as causal (monarch butterflies: Faldyn, Hunter, & Elderd, 2018; European honey bee: Kessler et al., 2015; Rundlöf et al., 2015). An estimate of the effects of the evolutionary trap effect associated with unpolarized street lamps in Germany indicated that the light could wipe out more than 60 billion insects over a single summer (Eisenbeis, 2006). Selection of narrow‐wavelength lamp types where safety and/or cost is allowed will lead to the most significant reductions in the attraction of aquatic (this study, Robertson, Campbell et al., 2017; Robertson, Ostfeld et al., 2017) and terrestrial (Pawson and Bader, 2014; Longcore et al., 2015) insects to nonhabitats, while more careful selection of potential artificial polarizers near water bodies (e.g., Horváth et al., 2010) will have an additional, though, minor benefit.

## CONFLICT OF INTEREST

We declare no competing interests.

## DATA ARCHIVING STATEMENT

Data are available from the Dryad Digital Repository: https://doi.org/10.5061/dryad.7m5cv66
